# Unpacking Data From Adult Day Centers in Order to Realize Their Untapped Potential in Dementia Care

**DOI:** 10.1093/geroni/igab046.318

**Published:** 2021-12-17

**Authors:** Tina Sadarangani, Lauren Parker

## Abstract

Adult day centers (ADCs) in the United States represent a vital, but commonly overlooked, resource for dementia care among community-dwelling older adults. However, the severity of dementia in ADC users, their medical complexity, the supports offered to them, and health outcomes associated with adult day services among persons living with dementia is poorly understood. This is in part due to a lack of standardized data collection in this industry. In this symposium, we present the most current research on these issues, as well as strategies to improve data collection across ADCs to strengthen care. The symposium begins with analysis of data from the state of California that identifies patterns of chronic conditions in ADC users with dementia that are associated with emergency department visits and hospitalizations. We then examine data from the Centers for Disease Control, comparing dementia specialized ADCs and their participants to non-specializing ADCs. We compare the extent to which states with ADC programs require collection of patient centered reported outcomes on persons with dementia. Finally, we explore an innovative collaboration between researchers and community partners to simplify data collection in these centers. Our findings suggest that persons with dementia in ADCs are an extremely complex population and that some ADCs are better suited than others to meet their extensive needs. Additional patient-centered data collection can be supported with widely available software, and has the potential to demonstrate the effectiveness of ADCs, aid in program development, and help leverage funding opportunities.

